# Health Effects of Power Loss After Hurricane Irma on Nursing Home Residents in Florida

**DOI:** 10.1093/geroni/igab046.1105

**Published:** 2021-12-17

**Authors:** Lily Gordon, Julianne Skarha, Nazmus Sakib, Joseph June, Dylan Jester, Lindsay Peterson, David Dosa

**Affiliations:** 1 Brown University, Providence, Rhode Island, United States; 2 Department of Industrial and Management Systems, University of South Florida, Tampa, Florida, United States; 3 University of South Florida, Tampa, Florida, United States; 4 University of California San Diego, La Jolla, California, United States; 5 University of South Florida, Bradenton, Florida, United States

## Abstract

Previous research establishes that hurricanes adversely affect nursing home (NH) resident health but specific causal pathways are still unclear. We combined power outage data with Medicare claims to determine the effects of power loss from Hurricane Irma(2017) among NH residents in Florida. Out of 580 facilities, 289 reported power loss. These facilities had higher star ratings; higher beds counts, and were preferentially in the Southeast region of Florida compared to facilities without outages. There were 27,767 residents living in a NH without power. They were comparable in characteristics to residents that did not lose power (N=26,383). We ran adjusted generalized linear models with robust standard errors, clustering for NH. We found power loss was associated with a trend towards increased odds of mortality within 7-days (OR:1.12, 95% CI:0.96, 1.30) and 30-days (OR:1.10, 95% CI:1.00, 1.21) post-storm, but not with hospitalization. Future research should investigate the time-specific effects of power outages.

